# Major Depressive Disorder and Oxidative Stress: A Review of Peripheral and Genetic Biomarkers According to Clinical Characteristics and Disease Stages

**DOI:** 10.3390/antiox12040942

**Published:** 2023-04-17

**Authors:** Abd El Kader Ait Tayeb, Vianney Poinsignon, Kenneth Chappell, Jérôme Bouligand, Laurent Becquemont, Céline Verstuyft

**Affiliations:** 1 Service de Génétique Moléculaire, Pharmacogénétique et Hormonologie de Bicêtre, Hôpitaux Universitaires Paris-Saclay, Assistance Publique-Hôpitaux de Paris, Hôpital de Bicêtre, F-94275 Le Kremlin Bicêtre, France; 2 CESP, MOODS Team, INSERM UMR 1018, Faculté de Médecine, Universitaires Paris-Saclay, F-94275 Le Kremlin Bicêtre, France; 3 INSERM UMR-S U1185, Faculté de Médecine, Universitaires Paris-Saclay, F-94275 Le Kremlin Bicêtre, France; 4 Centre de Recherche Clinique, Hôpitaux Universitaires Paris-Saclay, Assistance Publique-Hôpitaux de Paris, Hôpital de Bicêtre, F-94275 Le Kremlin Bicêtre, France

**Keywords:** major depressive disorder, antidepressant, clinical heterogeneity, oxidative stress, antioxidant defense

## Abstract

Major depressive disorder (MDD) is currently the main cause of disability worldwide, but its pathophysiology remains largely unknown, especially given its high heterogeneity in terms of clinical phenotypes and biological characteristics. Accordingly, its management is still poor. Increasing evidence suggests that oxidative stress, measured on various matrices such as serum, plasma or erythrocytes, has a critical role in MDD. The aim of this narrative review is to identify serum, plasma and erythrocyte biomarkers of oxidative stress in MDD patients according to disease stage and clinical features. Sixty-three articles referenced on PubMed and Embase between 1 January 1991, and 31 December 2022, were included. Modifications to antioxidant enzymes (mainly glutathione peroxidase and superoxide dismutase) in MDD were highlighted. Non-enzymatic antioxidants (mainly uric acid) were decreased in depressed patients compared to healthy controls. These changes were associated with an increase in reactive oxygen species. Therefore, increased oxidative damage products (principally malondialdehyde, protein carbonyl content and 8-hydroxy-2′-deoxyguanosine) were present in MDD patients. Specific modifications could be identified according to disease stages and clinical features. Interestingly, antidepressant treatment corrected these changes. Accordingly, in patients in remission from depression, oxidative stress markers were globally normalized. This narrative review suggests the particular interest of oxidative stress biomarkers for MDD care that may contribute to the heterogeneity of the disease and provide the opportunity to find new therapeutic targets.

## 1. Introduction

Major depressive disorder (MDD) is a common disease affecting more than 300 million people every year with a 12-month prevalence of 6% [1,2]. The lifetime prevalence is between 15% and 18%, ultimately affecting up to one in five individuals. Contrary to popular belief, it is equally common in high- and low-income countries, regardless of culture, origin or ethnicity [3]. Moreover, MDD is currently the main cause of disability worldwide, representing 7.5% of years lived with disability (YLD) across the globe at an estimated annual cost of over $US 1 trillion [2,4].

The DSM-5 describes a major depressive episode (MDE) as the combination of depressed mood and loss of interest or pleasure in almost all activities (anhedonia) with other symptoms such as weight change, sleep disturbances (insomnia or hypersomnia), psychomotor retardation (or agitation), fatigue, …, and suicidal ideation during the same 2-week period. These symptoms should cause clinically significant distress or social impairment and should not be attributable to a psychoactive substance or to another medical condition [5]. MDE are part of MDD or bipolar disorder (BP) if a history of (hypo-)mania is present. However, MDD cannot be reduced to these symptoms, as clinical heterogeneity is important. Indeed, depression may differ by symptom presentation (MDD with psychotic, melancholic, atypical, anxious, catatonic, or mixed features), severity, clinical course, age of onset, and response to treatment [6,7].

Unfortunately, despite its cost and prevalence, the treatment of MDD, which is essentially based on antidepressant drugs and psychotherapy, still has poor results [8]. Indeed, only one-third of patients achieve complete remission after a first-line antidepressant treatment, while one-third show no clinical response. Additionally, the cumulative complete remission rate reaches only 67% after four treatment steps with current therapeutic interventions [9,10]. Thus, it is mandatory to identify biomarkers of MDD that may help to improve disease prognosis [11].

One of the main issues regarding MDD is that the underlying mechanisms of the disorder remain largely unknown despite significant advances, likely contributing to the low effectiveness of care [1]. This lack of knowledge is due to multiple factors. First, the high clinical heterogeneity of MDD is likely associated with diverse pathophysiology, leading the National Institute of Mental Health (NIMH) to propose a new classification framework to improve research discoveries and clinical outcomes [7,12,13]. Second, the underlying mechanisms of MDD are associated with various biological pathways intertwined with psychological and social factors [14].

Among the different biological pathways, the monoaminergic hypothesis was the most explored. This theory emerged with observations of the mechanisms of action of the first antidepressant drugs. Nonetheless, this model failed to sufficiently explain MDD to effectively manage it [15,16]. Current data suggest that genetic, epigenetic (i.e., gene-environment interactions), and neuroendocrinological (e.g., hypothalamic-pituitary-adrenal axis changes) mechanisms are equally involved in MDD [1,15]. Additionally, inflammatory dysregulations (i.e., the inflammatory hypothesis of depression) and neuroanatomical modifications (e.g., affecting neuroplasticity and neurogenesis, inducing structural and functional brain changes) were also described to be related to the pathophysiology of MDD [1,15,17,18].

One of the main causes of neuroanatomical and inflammatory disturbances is oxidative stress [18,19,20]. Oxidative stress is the consequence of an accumulation of oxidative damage when antioxidant defenses fail to counteract the effects of free radical agents (reactive oxygen species (ROS) and reactive nitrogen species (RNS)). It is sometimes referred to as “oxidated distress” as opposed to “oxidative eustress” since free radical agents are involved in physiological processes [20,21]. Indeed, under normal conditions, ROS and RNS are implicated in many biological pathways secondary to redox signaling (e.g., cellular growth, survival, and regulation) [21,22]. However, oxidative stress impairments are commonly described in disease pathophysiology. Interestingly, the brain is more vulnerable to oxidative stress due to its high metabolic rate and lower antioxidant levels [18,21]. Therefore, growing evidence shows the involvement of oxidative stress in brain-related diseases, such as Alzheimer’s disease, schizophrenia, and MDD [19,20,23].

Hence, the first aim of this narrative review is to consolidate current data on peripheral and genetic biomarkers of oxidative stress in MDD, particularly in relation to its clinical features. The second aim is to provide a better understanding of pathophysiological processes underlying unipolar depression.

## 2. Methods

The literature was reviewed by an extensive search of studies published between 1 January 1990, and 31 December 2022. The extensive search was conducted in two databases, PubMed/Medline and Embase.

For metabolomic parameters, inclusion and exclusion criteria were adapted from Rambaud et al. [23] for use with MDD. The keywords used for the search were: (“depression” OR “major depression” OR “major depressive disorder” OR “major depressive episode”) AND (“antioxidant” OR “oxidative stress”). The inclusion criteria for the article were: only published in peer-reviewed journals; published in English; patients diagnosed with MDD according to the Diagnostic and Statistical Manual of Mental Disorders (DSM) or the International Classification of Disease (ICD); studies including healthy controls; studies performed in humans; articles could be clinical studies, clinical trials, case reports, comparative studies, meta-analyses, or multicenter studies. Exclusion criteria were: studies exploring a specific biomarker that is not commonly investigated for oxidative stress; tissues other than serum, plasma or erythrocytes were studied (since oxidative stress may have contrasting effects in different tissues). The results from studies were presented separately for each matrix, as the identified changes may be different for each of them. For genetic parameters, the keywords used were: (“depression” OR “major depression” OR “major depressive disorder” OR “major depressive episode”) AND (“antioxidant” OR “oxidative stress”) AND (“polymorphism”). The inclusion and exclusion criteria were similar, but all types of articles were included here. Lastly, supplemental studies in the bibliographies of selected articles could be included in this review.

**Figure 1:**
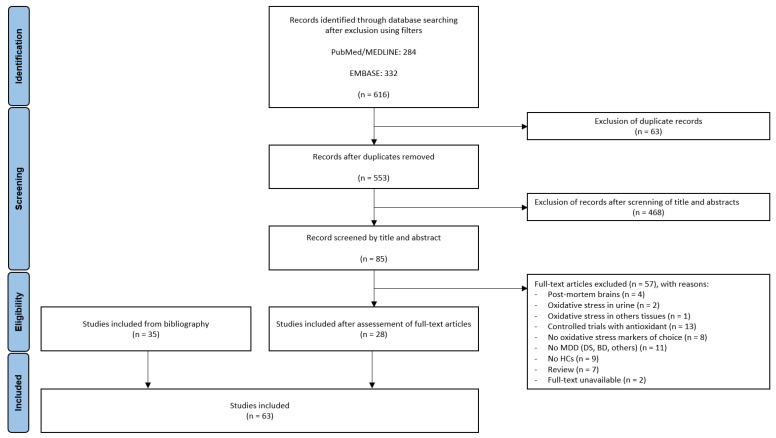
**The PRISMA flowchart**.

The screening of studies was performed independently by two investigators (AEKAT and VP). In case of disagreement, the relevance of the study was assessed by both investigators. The PRISMA flowchart describing the different stages of the literature search and inclusion and exclusion for each stage is shown in Figure 1.

## 3. Results

In total, 616 articles (284 from PubMed and 332 from Embase) were identified. After removing duplicate records, 553 publications were screened by their titles and abstracts. Following this first screening, 85 full-text articles were assessed for eligibility. Among them, 28 were relevant according to the criteria. Thirty-five supplemental articles identified in the bibliographies were added. Thus, 63 studies were included in this narrative review. The selected articles were comparative studies between healthy subjects and patients suffering from an MDE in the context of MDD, according to disease stage and clinical features. A summary of the biomarkers identified from the 63 articles is provided in Table 1, Table 2, Table 3, Table 4, Table 5, Table 6, Table 7, Table 8 and Table 9. Biomarkers were grouped according to their nature (e.g., antioxidant enzymes, non-enzymatic antioxidants, ROS-producing enzymes, ROS/RNS, oxidative damage products and others), the examined biological matrix (i.e., serum, plasma, erythrocytes), and clinical characteristics.

**Figure 2:**
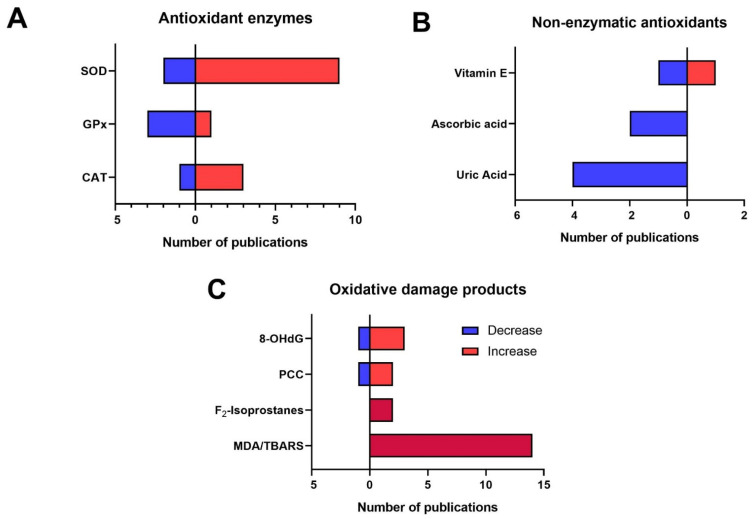
**Number of publications according to modifications evidenced in main peripheral biomarkers of oxidative stress in unspecified MDD patients compared to healthy controls.** MDD: Major depressive disorder—CAT: Catalase—GPx: Glutathion Peroxydase—SOD: Superoxide dismutase (information about isoform was not provided)—MDA: Malondialdehyde—TBARS—Thiobarbituric Acid-Reactive Substances—PCC: Protein carbonyl content—8-OHdG: 8-hydroxy-2′-deoxyguanosine. (**A**) According to the antioxidant enzymes. (**B**) According to the non-enzymatic antioxidants. (**C**) According to the oxidative damage products.

Among the 63 articles, 39 did not provide enough specific clinical information to specifically characterize the depressive episode. They were qualified as “unspecified MDD”. Globally, in depressed patients, an oxidative stress imbalance was observed with changes in antioxidant enzymes, decreases in non-enzymatic antioxidants, increases in ROS and RNS, and, accordingly, increases in oxidative damage products of DNA, lipids, and proteins (see Table 1 and Figure 2). Detailed results are discussed further.

For clinical features, three different clinical contexts were identified in five articles: melancholic features, psychotic features, and suicidal symptoms (i.e., suicide attempt or suicidal ideation) (see Table 2). Although biomarker modifications were also observed in patients without melancholic features compared to healthy controls, more modifications with greater variability were observed in patients with melancholic symptoms compared to healthy controls. Similarly, more changes were observed in patients with suicidal symptoms than without. For MDD with psychotic features, no differences were highlighted between depressed patients and healthy subjects.

Three different stages of MDD were also identified: first depressive episode (see Table 3), recurrent MDD (see Table 4), and MDD in remission (see Table 5). Six articles described modifications in the first MDE, revealing a decrease in antioxidant capacity and an increase in two biomarkers associated with oxidative damage (see Table 3). Modifications in recurrent MDD were described in four articles and showed a higher level of ROS, lower levels of antioxidant enzymes, and a higher level of oxidative damage (see Table 4). Concerning the three articles describing MDD in remission, no clear difference between patients and healthy controls was observed (see Table 5).

Oxidative stress biomarkers were also assessed according to the severity of MDD in five articles (see Table 6). All groups of depressed patients (i.e., mild, moderate, and severe MDD) showed increases in antioxidant enzymes and oxidative damage products compared to healthy controls. With respect to the age of MDD onset, two groups were described in five articles: child and adolescent depression and geriatric depression (see Table 7). In child and adolescent depression, an increased total oxidant status (TOS) and a decreased total antioxidant capacity (TAC) were observed. For geriatric depression, lower antioxidant activities associated with higher levels of ROS and oxidative damage products were revealed.

**Table 1:** **Peripheral biomarkers of oxidative stress in unspecified MDD patients compared to healthy controls**. MDD: Major depressive disorder—CAT: Catalase—GPx: Glutathion Peroxydase—SOD: Superoxide dismutase (information about isoform was not provided)—Cu/ZnSOD: Type 1 isoform of SOD—GSSG: Glutathione disulfide—GSH: Reduced form of glutathione—XO: Xanthine oxidase—H_2_O_2_: Hydrogen peroxide—NO: Nitric oxide—MDA: Malondialdehyde—TBARS—Thiobarbituric Acid-Reactive Substances—oxLDL: Oxide LDL—PCC: Protein carbonyl content—8-OHdG: 8-hydroxy-2′-deoxyguanosine—TAC: Total antioxidant capacity– TOS: Total oxidant status—↗: Values significantly higher in depressed patients compared to controls—↘: Values significantly lower in depressed patients compared to controls—↔: No significant difference between depressed patients and controls.

Biomarkers	Biological Matrix	Modification in MDD Patients
**Antioxidant enzymes**		
CAT	Serum	↗ [24,25]
	Erythrocytes	↗ [26], ↘ [27]
GPx	Serum	↗ [24], ↘ [28], ↔ [25]
	Plasma	↔ [29,30]
	Erythrocytes	↘ [27], ↔ [26,31]
	Whole blood	↘ [32], ↔ [33]
SOD	Serum	↗ [24,25,34,35], ↘ [28,36,37], ↔ [38,39]
	Plasma	↔ [29]
	Erythrocytes	↗ [33], ↔ [27]
Cu/ZnSOD	Serum	↗ [40]
	Erythrocytes	↗ [26,31]
**Non-enzymatic antioxidants**		
GSSG	Plasma	↔ [30]
GSH	Plasma	↔ [30]
Uric acid	Serum	↘ [41,42,43,44], ↔ [31,45,46,47]
	Whole blood	↔ [33]
Ascorbic acid	Serum	↘ [36], ↔ [30,31]
	Plasma	↘ [34], ↔ [33]
Vitamin E	Serum	↘ [48], ↔ [31]
	Plasma	↗ [33]
Thiols	Plasma	↗ [49]
**ROS/RNS-producing enzymes**		
XO	Serum	↗ [37]
NO synthase	Serum	↗ [35]
	Plasma	↘ [50]
**Reactive oxygen species (ROS) and reactive nitrogen species (RNS)**		
H_2_O_2_	Plasma	↗ [51]
NO	Serum	↗ [39], ↔ [37]
	Erythrocytes	↘ [27]
**Oxidative damage products**		
MDA/TBARS	Serum	↗ [28,34,36,38,39,52,53], ↔ [25,35,45,47,54,55]
	Plasma	↗ [29,31,33,56]
	Erythrocytes	↗ [26,27,33]
F_2_-Isoprostanes	Serum	↗ [57], ↔ [58]
	Plasma	↗ [30]
oxLDL	Serum	↗ [51], ↔ [31]
PCC	Serum	↗ [54,55], ↘ [25], ↔ [45,47]
8-OHdG	Serum	↗ [59], ↘ [58], ↔ [25]
	Plasma	↗ [30,60]
**Others**		
TAC	Serum	↘ [33,52,61]
	Plasma	↘ [44,62]
	Erythrocytes	↘ [26]
TOS	Serum	↗ [61]
	Plasma	↗ [62]

**Table 2:** **Peripheral biomarkers of oxidative stress in MDD patients according to clinical features compared to healthy controls.** MDD: Major depressive disorder—CAT: Catalase—GPx: Glutathion Peroxydase—GR: Glutathion Reductase—SOD: Superoxide dismutase (information about isoform was not provided)—GSH: Reduced form of glutathione—MDA: Malondialdehyde—TBARS—Thiobarbituric Acid-Reactive Substances—PCC: Protein carbonyl content—TAC: Total antioxidant capacity—↗: Values significantly higher in depressed patients compared to controls—↘: Values significantly lower in depressed patients compared to controls—↔: No significant difference between depressed patients and controls.

Biomarkers	Biological Matrix	Modification in MDD
MDD with melancholic features		
**Antioxidant enzymes**		
CAT	Erythrocytes	↔ [63]
GPx	Plasma	↔ [63]
	Erythrocytes	↗ [63]
GR	Plasma	↗ [63]
	Erythrocytes	↔ [63]
SOD	Erythrocytes	↗ [63]
**Oxidative damage products**		
MDA	Plasma	↗ [63]
	Erythrocytes	↗ [63]
TBARS	Serum	↗ [53], ↔ [54]
PCC	Serum	↗ [54]
**MDD without melancholic characteristics**		
**Antioxidant enzymes**		
CAT	Erythrocytes	↔ [63]
GPx	Plasma	↔ [63]
	Erythrocytes	↔ [63]
GR	Plasma	↔ [63]
	Erythrocytes	↔ [63]
SOD	Erythrocytes	↗ [63]
**Oxidative damage products**		
MDA	Plasma	↗ [63]
	Erythrocytes	↗ [63]
TBARS	Serum	↔ [54]
PCC	Serum	↗ [54]
**MDD with suicidal symptoms**		
**Non-enzymatic antioxidants**		
Uric acid	Serum	↘ [64]
**Oxidative damage products**		
TBARS	Serum	↗ [53]
**MDD without suicidal symptoms**		
**Non-enzymatic antioxidant**		
Uric acid	Serum	↔ [64]
**MDD with psychotic features**		
**Antioxidant enzymes**		
GPx	Erythrocytes	↔ [65]
**Non-enzymatic antioxidants**		
GSH	Erythrocytes	↔ [65]
**Oxidative damage products**		
LOOH	Plasma	↔ [65]
**Others**		
TAC	Plasma	↔ [65]

**Table 3:** **Peripheral biomarkers of oxidative stress in patients with a first major depressive episode compared to healthy controls.** MDE: Major depressive episode—CAT: Catalase—GPx: Glutathion Peroxydase—GST: Glutathion-S-Transferase—SOD: Superoxide dismutase (information about isoform was not provided)—Cu/ZnSOD: Type 1 isoform of SOD—MDA: Malondialdehyde—8-OHdG: 8-hydroxy-2′-deoxyguanosine—↗: Values significantly higher in depressed patients compared to controls—↘: Values significantly lower in depressed patients compared to controls—↔: No significant difference between depressed patients and controls.

Biomarkers	Biological Matrix	Modification in First MDE
**Antioxidant enzymes**		
CAT	Erythrocytes	↔ [66]
GPx	Serum	↔ [28]
GST	Serum	↔ [67]
SOD	Serum	↘ [28], ↔ [38]
	Plasma	↔ [67]
	Erythrocytes	↘ [66]
Cu/ZnSOD	Plasma	↗ [68]
**Oxidative damage products**		
MDA	Serum	↔ [28]
	Plasma	↗ [66], ↔ [67]
8-OHdG	Serum	↗ [59]

**Table 4:** **Peripheral biomarkers of oxidative stress in recurrent MDD patients compared to healthy controls.** rcMDD: Recurrent major depressive disorder—GPx: Glutathion Peroxydase—GR: Glutathion Reductase—SOD: Superoxide dismutase (information about isoform was not provided)—Cu/ZnSOD: Type 1 isoform of SOD—MnSOD: Type 2 isoform of SOD—GSH: Reduced form of glutathione—H_2_O_2_: Hydrogen peroxide—MDA: Malondialdehyde—8-OHdG: 8-hydroxy-2′-deoxyguanosine—↗: Values significantly higher in depressed patients compared to controls—↘: Values significantly lower in depressed patients compared to controls—↔: No significant difference between depressed patients and controls.

Biomarkers	Biological Matrix	Modification in rcMDD
**Antioxidant enzymes**		
GPx	Serum	↘ [28]
	Erythrocytes	↘ [69]
GR	Erythrocytes	↗ [69]
SOD	Serum	↘ [28]
Cu/ZnSOD	Erythrocytes	↘ [69]
MnSOD	Serum	↘ [70]
**Non-enzymatic antioxidants**		
GSH	Whole blood	↗ [69]
**Reactive oxygen species (ROS)**		
H_2_O_2_	Plasma	↗ [69]
**Oxidative damage products**		
MDA	Serum	↗ [28]
	Erythrocytes	↗ [69]
8-OHdG	Serum	↗ [59]

**Table 5:** **Peripheral biomarkers of oxidative stress in MDD patients in remission compared to healthy controls**. rMDD: Major depressive disorder in remission—GPx: Glutathion Peroxydase—SOD: Superoxide dismutase (information about isoform was not provided)—MDA: Malondialdehyde—TBARS—Thiobarbituric Acid-Reactive Substances—8-OHdG: 8-hydroxy-2′-deoxyguanosine—↗: Values significantly higher in depressed patients compared to controls—↘: Values significantly lower in depressed patients compared to controls—↔: No significant difference between depressed patients and controls.

Biomarkers	Biological Matrix	Modification in rMDD
**Antioxidant enzymes**		
GPx	Plasma	↔ [29]
SOD	Plasma	↔ [29]
**Non-enzymatic antioxidants**		
Uric acid	Serum	↘ [71]
**Oxidative damage products**		
MDA/TBARS	Plasma	↔ [29]
F_2_-Isoprostanes	Plasma	↔ [58]
8-OHdG	Plasma	↔ [58]

**Table 6:** **Peripheral biomarkers of oxidative stress according to severity of MDD patients compared to healthy controls.** MDD: Major depressive disorder—GR: Glutathion Reductase—SOD: Superoxide dismutase (information about isoform was not provided)—MDA: Malondialdehyde—oxLDL: Oxide LDL—8-OhdG: 8-hydroxy-2′-deoxyguanosine—↗: Values significantly higher in depressed patients compared to controls—↘: Values significantly lower in depressed patients compared to controls—↔: No significant difference between depressed patients and controls.

Biomarkers	Biological Matrix	Modifications in MDD		
		Mild MDD	Moderate MDD	Severe MDD
**Antioxidant enzymes**				
GR	Plasma	↗ [72]	↗ [72]	↗ [72]
SOD	Erythrocytes	↗ [33]	↗ [33]	↗ [33]
**ROS/RNS-producing enzymes**				
NO synthase	Plasma	↗ [73]	↗ [73]	↗ [73]
**Oxidative damage products**				
MDA	Serum	↗ [74]	↗ [74]	↗ [74]
	Plasma	↗ [33]	↗ [33]	↗ [33]
	Erythrocytes	↗ [33]	↗ [33]	↗ [33]
OxLDL	Plasma	↗ [72]	↗ [72]	↗ [72]
8-OhdG	Serum	↔ [59]		↗ [59]

**Table 7:** **Peripheral biomarkers of oxidative stress in MDD patients according to the disorder onset compared to healthy controls.** MDD: Major depressive disorder—GPx: Glutathion Peroxydase—GR: Glutathion Reductase—GST: Glutathion-S-Transferase—SOD: Superoxide dismutase (information about type was not provided)—GSH: Reduced form of glutathione—NO: Nitric oxide—MDA: Malondialdehyde—TBARS—Thiobarbituric Acid-Reactive Substances—PCC: Protein carbonyl content—TAC: Total antioxidant capacity—TOS: Total oxidant status—↗: Values significantly higher in depressed patients compared to controls—↘: Values significantly lower in depressed patients compared to controls—↔: No significant difference between depressed patients and controls.

Biomarkers	Biological Matrix	Modification in MDD
Child and adolescent depression		
**Antioxidant enzymes**		
SOD	Serum	↘ [75]
**Non-enzymatic antioxidants**		
Uric acid	Serum	↗ [76]
**Oxidative damage products**		
MDA	Serum	↗ [75]
**Others**		
TAC	Serum	↘ [75]
TOS	Serum	↗ [75]
**Geriatric depression**		
**Antioxidant enzymes**		
GPx	Plasma	↘ [77]
GR	Plasma	↔ [77]
GST	Plasma	↔ [77]
SOD	Serum	↘ [78]
**Non-enzymatic antioxidants**		
GSH	Serum	↘ [78]
**ROS & RNS**		
NO	Serum	↗ [78]
**Oxidative damage products**		
F_2_-Isoprostanes	Plasma	↗ [77,79]
TBARS	Plasma	↔ [77]
PCC	Serum	↗ [78]
	Plasma	↔ [77]

Of note, among the 63 studies, 18 realized pre- and post-treatment follow-up of patients and described changes in biomarkers of oxidative stress after an antidepressant treatment in patients with unipolar depression, while the other studies were only cross-sectional studies. Results in Table 8 described parameter values in depressed patients before and after treatment, independently of clinical features and stage of the disorder (see Table 8). Thus, no clear difference in antioxidant enzymes was observed. As well, increases in non-enzymatic antioxidants were found. Reduced levels of ROS and oxidative damage products were also identified in depressed patients after antidepressant treatment compared to before.

**Table 8:** **Evolution of peripheral biomarkers of oxidative stress in MDD patients after antidepressant treatment**. MDD: Major depressive disorder—CAT: Catalase—GPx: Glutathion Peroxydase—GR: Glutathion Reductase—GST: Glutathion-S-Transferase—SOD: Superoxide dismutase (information about isoform was not provided)—Cu/ZnSOD: Type 1 isoform of SOD—GSSG: Glutathione disulfide—GSH: Reduced form of glutathione—XO: Xanthine oxidase—H_2_O_2_: Hydrogen peroxide—NO: Nitric oxide—MDA: Malondialdehyde—TBARS—Thiobarbituric Acid-Reactive Substances—oxLDL: Oxide LDL—PCC: Protein carbonyl content—8-OHdG: 8-hydroxy-2′-deoxyguanosine—TAC: Total antioxidant capacity—TOS: Total oxidant status—↗: Values significantly higher in depressed patients after treatment compared to before—↘: Values significantly lower in depressed patients after treatment compared to before—↔: No significant difference between depressed patients after treatment compared to before.

Biomarkers	Biological Matrix	Modification after Treatment
**Antioxidant enzymes**		
CAT	Serum	↔ [25]
	Erythrocytes	↔ [26,27,63]
GPx	Serum	↘ [28], ↔ [25]
	Plasma	↘ [63], ↔ [30]
	Erythrocytes	↗ [27], ↔ [26,31,63]
	Whole blood	↔ [33]
GR	Plasma	↘ [63]
	Erythrocytes	↔ [63]
GST	Serum	↔ [67]
SOD	Serum	↗ [37], ↘ [28,34], ↔ [25,67]
	Erythrocytes	↘ [31,63], ↔ [27,33]
Cu/ZnSOD	Erythrocytes	↔ [26]
**Non-enzymatic antioxidants**		
GSSG	Plasma	↔ [30]
GSH	Plasma	↔ [30]
Uric acid	Serum	↗ [41], ↔ [31]
	Whole blood	↔ [33]
Ascorbic acid	Serum	↔ [30]
	Plasma	↗ [34], ↔ [31,33]
Vitamin E	Plasma	↔ [31,33]
**ROS-producing enzymes**		
XO	Serum	↘ [37]
NO synthase	Plasma	↗ [50]
**Reactive oxygen species (ROS) and reactive nitrogen species (RNS)**		
NO	Serum	↘ [37]
**Oxidative damage products**		
MDA/TBARS	Serum	↘ [34], ↗ [28], ↔ [25,52]
	Plasma	↘ [63], ↔ [33,67]
	Erythrocytes	↘ [26,63], ↔ [27,33]
F_2_-Isoprostanes	Plasma	↔ [30,58]
oxLDL	Serum	↔ [31]
PCC	Serum	↔ [25]
8-OHdG	Serum	↔ [25]
	Plasma	↘ [58,60], ↗ [30]
**Others**		
TAC	Serum	↗ [61], ↔ [52]
	Plasma	↔ [26,33,62]
TOS	Serum	↘ [61]
	Plasma	↔ [62]

Finally, concerning the genetics of enzymes involved in oxidative stress, eight polymorphisms (two of *SOD2*, one of *GPX1*, one of *GPX4*, two of *CAT*, one of *GSTM1* and one of *GSTT1)* were assessed in seven articles (see Table 9). Among them, one of *SOD2* (rs4880), one of *CAT* (rs7943316), and those of *GPX1*, *GPX4*, *GSTM1*, and *GSTT1* were described as associated with MDD prevalence. Nonetheless, most of these results have not been replicated in independent cohorts.

**Table 9:** **Associations between genetic polymorphisms of antioxidant enzymes and the prevalence of MDD.** MDD: Major depressive disorder—*CAT*: coding for catalase—*GPX:* coding for glutathione peroxidase—*GST*: coding for glutathione-S-Transferase—*SOD2*: coding for MnSOD.

Genes	Genetic Polymorphisms	Associations with MDD		
		Protective Effect	Risk Factor	No Association
*CAT*	rs7943316		x [80]	
	rs1001179			x [81]
*GPX1*	rs1050450	x [82]		
*GPX4*	rs713041	x [80]		
*GSTM1*	Gene deletion	x [83]		x [84]
*GSTT1*	Gene deletion	x [83]		x [84]
*SOD2*	rs4880	x [80,85]		x [82,86]
	rs1141718			x [85]

## 4. Discussion

### 4.1. Biomarkers of Oxidative Stress in MDD

From 616 initial articles, this narrative review identified 63 studies that explored associations between biomarkers related to oxidative stress and MDD. Among these biomarkers, several appear to be potential candidates for the management of MDD. Here, we discuss peripheral biomarkers of oxidative stress in unspecified MDD and compare them with brain data from postmortem studies.

#### 4.1.1. Reactive Oxygen Species and Reactive Nitrogen Species

First, modifications in ROS and RNS concentrations were found. An increase in plasma hydrogen peroxide (H_2_O_2_) was associated with MDD. Changes in nitric oxide (NO) were less consistent, with increased and decreased concentrations in serum and erythrocytes, respectively [27,37,39,51].

#### 4.1.2. Antioxidant Enzymes

Second, antioxidant enzymes were largely explored. Superoxide dismutases (SODs) are the first and main protective barrier against oxidative stress [87,88]. These enzymes catalyze the dismutation reaction of O_2_^−^ (principally produced by the mitochondrial respiratory chain) in H_2_O_2_ [88]. Three isoforms of SOD exist: Cu/ZnSOD (coded by *SOD1*) located in the cytosol and nucleus; MnSOD (coded by *SOD2*) located in mitochondria; ECSOD (coded by *SOD3*) located in the extracellular medium [89]. Several studies did not specify which SOD isoforms were assessed. If available, information about SOD isoforms was provided in the tables. Regarding SOD expression and activity in serum, plasma, or erythrocytes, studies were highly contradictory. Several showed increases in SOD expression and activity in serum and erythrocytes [24,25,26,31,33,34,35,40], but three showed decreases [28,36,37] and four reported no significant differences [27,29,38,39]. Hence, the last meta-analysis performed failed to identify a clear trend of SOD activity in MDD [90]. Results were controversial in postmortem studies, as well. Cu/ZnSOD expression appeared to be increased in the prefrontal cortex and decreased in the occipital cortex and brainstem, while no change was observed in the hippocampus [91,92]. For MnSOD, similar results were found [91,93]. Concerning genetic polymorphisms, the *SOD2 Val16Ala* polymorphism (rs4880) was described to be associated with MDD prevalence [87]. In two studies, the *Ala16* allele seemed to be a protective factor in MDD [80,85]. Remarkably, in a genetic study performed on a general population, this allele was associated with a higher risk for depressive symptoms [94]. This allele is associated with increased enzymatic activity compared to the *Val16* allele [88]. Nonetheless, these results were controversial since two other studies failed to show any association with MDD [82,86]. Of note, the positive genetic studies were performed in different ethnic groups compared to negative studies. To the best of our knowledge, until now, no study has assessed the association between *SOD1* and *SOD3* genetic polymorphisms and MDD.

After the dismutation reaction of superoxide anion, hydrogen peroxide is metabolized by catalase (coded by *CAT*) or by glutathione peroxidase (GPx) [95]. Catalase is responsible for the dismutation reaction of H_2_O_2_ to O_2_ and H_2_O. It is mainly localized in the peroxisome, but it has also been detected in the cytoplasm and mitochondria [96]. In unspecified MDD, four studies were identified. Globally, catalase activity was increased in depressed patients compared to healthy controls. More specifically, three studies demonstrated increased CAT activity in serum [24,25] and erythrocytes [26]. However, decreased activity was also described in erythrocytes [27]. In a meta-analysis, catalase activity was increased in MDD patients [90]. This increased catalase activity could be a compensatory mechanism to reduce ROS concentrations in MDD. In genetic studies, two polymorphisms of *CAT* (rs7943316 and rs1001179) were explored in Caucasian cohorts of MDD, but only rs7943316 was associated with MDD [80,81]. However, the mechanism of this association remains unknown since this genetic polymorphism is not located in a coding region of *CAT*.

H_2_O_2_ can also be metabolized by GPx. At least eight different isoforms of GPx have been identified in humans with various localizations. For example, GPx3 is found in mitochondria [22]. Each GPx is coded by a different gene (from *GPX1* to *GPX8*). The metabolism of H_2_O_2_ to H_2_O and glutathione disulfide (GS-SG) by GPx requires the reduced form of glutathione (GSH) as a cofactor. Among the ten studies found in unspecified MDD, three described decreased GPx concentrations or activities [27,28,32], one an increase [24], and six no significant differences [25,26,29,30,31,33]. However, in a meta-analysis, GPx were described as reduced in peripheral samples of depressed patients compared to healthy controls [90]. Additionally, in a postmortem study, GPx levels were decreased in the prefrontal cortex of MDD patients [97]. Two genetic polymorphisms of GPx in two different genes, *GPX1* and *GPX4*, were associated with MDD [80,82]. Of note, rs1050450 of *GPX1* was also associated with depressive symptoms in the general population [98]. Notably, peripheral GPx levels could be particularly useful since they can distinguish an MDE in the context of MDD from an MDE in the context of BP. Indeed, no significant difference in GPx levels between BP and controls was observed [90].

#### 4.1.3. Non-Enzymatic Antioxidants

Third, in our narrative review, many changes in non-enzymatic antioxidants were described. In unspecified depression, no modification of glutathione was observed in peripheral samples, either in its reduced (GSH) or oxidized/disulfide form (GS-SG) [30]. GSH, the main cell antioxidant compound, is a tripeptide used as a cofactor by GPx during the detoxification of H_2_O_2_. GSH levels are restored by glutathione reductase (GR), which reduces GS-SG to two GSH. GSH is also involved in the mechanism of glutathione-S-transferase (GST), which metabolizes xenobiotics [22]. In a postmortem study of the prefrontal cortex, decreased levels of GSH and GS-SG were associated with MDD [97]. GST is coded by several genes. Among them, a deletion in *GSTM1* and *GSTT1* appeared to be a protective factor for MDD [83], but this result was not replicated [84].

Another largely described antioxidant is uric acid. Despite its antioxidant effect, this low-molecular-weight organic compound from the purine degradation pathway is associated with several disorders in the context of hyperuricemia, including gout and arterial hypertension [99]. In serum, four studies identified a lower level of uric acid in patients suffering from an unspecified MDD compared to controls [41,42,43,44], though four observed no significant differences [31,45,46,47]. In whole blood, there was no modification between patients and controls [33]. In concordance with these studies, two large populational cohorts, representing 96,989 individuals, observed higher levels of plasma uric acid that were associated with a reduced risk of hospitalization for depression and a reduced risk to receive an antidepressant medication [100]. Similar to GPx, uric acid could be a useful biomarker of MDD, as it also allows us to discriminate an MDE in the context of MDD from one in the context of BP [42,43,71,90].

Ascorbic acid, also known as vitamin C, is a critical molecule for various biological pathways from collagen synthesis to neuroprotection [22,101]. Even if its mechanisms remain partially unknown, ascorbic acid is also described as an antioxidant agent [101]. In our screening, two studies in serum and plasma described lower levels of ascorbic acid in MDD patients [34,36] although these results were not systematically replicated [30,31,33].

Vitamin E is well-known as an antioxidant agent. In unspecified MDD, case-control studies exploring vitamin E concentrations were highly inconsistent. Indeed, of the three identified studies, one showed an increase, one a decrease, and one no significant difference [31,33,48]. In a Japanese cohort, a high level of professional stress that was associated with depressive symptoms also identified reduced levels of vitamin E [102]. In a large Dutch cohort of 721 individuals, men had a lower level of vitamin E if they suffered from depressive symptoms. Surprisingly, this result was not replicated in women [103].

#### 4.1.4. Oxidative Damage Products

Lastly, numerous variations in oxidative damage products were found in depressed patients compared to healthy controls. Foremost, lipid oxidative damage was identified, as revealed by changes in concentrations of malondialdehyde (MDA), thiobarbituric acid-reactive substances (TBARS), F_2_-isoprostanes, and oxide LDL (oxLDL). Concerning these biomarkers, MDA is a byproduct of polyunsaturated fatty acid peroxidation, which is commonly used as a measure of lipid peroxidation due to oxidative stress. TBARS is also used to quantify MDA, and F_2_-isoprostanes are a classic biomarker of lipid peroxidation [18,104]. Compared to healthy subjects, nine publications identified an increase in lipid oxidative damage in the serum of MDD patients [28,34,36,38,39,51,52,53,57], five in plasma [29,30,31,33,56], and three in erythrocytes [26,27,33]. Of note, eight studies failed to identify any difference [25,31,35,45,47,54,55,58]. In MDD patients compared to controls, levels of F_2_-isoprostanes in cerebrospinal fluid were higher in depression [105]. Additionally, F_2_-isoprostanes levels were higher in the urine of depressed patients [106], whereas no difference was observed in peripheral blood mononuclear cells (PBMC) [107]. In the general population, elevated plasma F_2_-isoprostanes levels were associated with depressive symptoms [108]. In two different meta-analyses, TBARS and F_2_-isoprostanes levels were increased in depression and depressive symptoms [90,109].

Proteins also undergo oxidative damage in unipolar depression. By far, the biomarker of protein oxidation classically used is protein carbonyl content (PCC) [110]. In association with MDD, serum levels of PCC were higher in two articles [54,55], lower in one article [25], and not different in two articles [45,47]. A meta-analysis of five articles failed to show a significant difference between PCC levels in depressed patients and controls [111].

Similar to lipids and proteins, oxidative damage also affects DNA in MDD. One of the most common biomarkers of DNA oxidative damage is 8-hydroxy-2′-deoxyguanosine (8-OHdG), which is the final product of guanine hydroxylation [19,22]. Among three articles investigating serum 8-OHdG levels in depressed patients with respect to healthy subjects, one found increased levels of 8-OHdG [59], one decreased levels [58], and one no difference [25]. In plasma, MDD was associated with higher levels of 8-OHdG in both publications [30,60]. In a postmortem study, an increase in DNA oxidation (estimated by 8-OHdG) in brain white matter was observed in MDD patients [93]. In urine, 8-OHdG appeared to be increased in the context of MDD and depressive symptoms, but the results were less consistent [67,112,113]. 8-OHdG was also increased in leukocytes of depressed patients compared to healthy individuals [114]. In a meta-analysis, DNA oxidation damage seem to be higher in MDD patients compared to controls [109]. Interestingly, modifications of urinary 8-oxo-7,8-dihydroguanosine (8-oxoGuo), a biomarker of RNA oxidation, were also observed in MDD [115,116]. Increased hippocampal RNA oxidation was also observed in a postmortem study of depressed patients [117].

#### 4.1.5. Others

Among other oxidative stress biomarkers, xanthine oxidase (XO) is another example. XO is a major ROS-producing enzyme, notably of superoxide anion and hydrogen peroxide [118]. In this review, we identified one article that described higher XO activity in depressed patients compared to controls [37]. In a postmortem study, XO activity was also increased in the putamen and thalamus of depressed patients compared to healthy subjects [118]. As well, changes in NO synthase, the main source of NO in an organism, appeared, in two articles, with a decreased plasma activity in MDD patients compared to healthy subjects while an increase was evidenced in serum [22,35,50].

Of note, as previously described, values of different antioxidant molecules and oxidant species can be measured separately. However, since their effects are additive, measures grouping antioxidant and oxidant activities may be used to simplify analyses [119]. Hence, TAC provides information about the cumulative effect of all antioxidants in the analyzed biological matrix [120]. Similarly, TOS provides information about the effect of all oxidants [119]. In the screened articles, three publications in serum [33,52,61], two in plasma [44,62], and one in erythrocytes [26] identified reduced TAC in MDD patients compared to healthy controls. In a meta-analysis, peripheral TAC in depressed patients appeared to be lower compared to controls [111]. Similar results were found in a general population based on depressive symptoms [121]. For TOS, two publications in serum and plasma showed increased levels in depressed patients [61,62].

### 4.2. Oxidative Stress Imbalance, a Pathway to Explain MDD Heterogeneity?

MDD is a disorder with a high level of clinical and biological heterogeneity. In this section, differences in oxidative stress biomarkers are discussed in relation to clinical features and disease stages.

#### 4.2.1. MDD with Melancholic Features

Changes in oxidative stress have been demonstrated in MDD with or without melancholic features (see Table 2). Unlike patients suffering from MDD without melancholic features, patients with melancholic features had a greater activity of GPx (in erythrocytes) and GR (in plasma) compared to healthy subjects [63]. Additionally, higher serum TBARS levels were observed in melancholic MDD compared to healthy controls [53]. There was no difference in the activity of CAT and SOD, nor in MDA and PCC levels, between patients with and without melancholic features compared to controls [54,63]. More changes related to oxidative stress in melancholic patients were not unexpected, since MDD with melancholic features is generally considered more severe than MDD without melancholic features. Nonetheless, results for melancholia should be interpreted with caution since the sample sizes of the different studies were small.

#### 4.2.2. MDD with Suicidal Symptoms

Few data specifically exploring oxidative stress in MDD according to the presence of suicidal symptoms were identified in our bibliographic research. Patients with MDD associated with suicidal symptoms had lower levels of serum uric acid compared to healthy controls, whereas no differences were observed in patients without suicidal symptoms [64]. An increase in lipid peroxidation (TBARS) was also observed in suicidal patients compared to controls [53]. In two meta-analyses, suicidal ideation and suicide attempt were associated with higher levels of oxidative stress, independent of clinical context [122,123]. As for melancholic depression, MDD with suicidal symptoms is judged as more severe, which may explain the observed difference.

#### 4.2.3. MDD with Psychotic Symptoms

For psychotic depression (see Table 2), no differences in antioxidant enzymes (GPx), non-enzymatic antioxidants (GSH), lipid peroxidation, or TAC in erythrocytes or plasma were observed between depressed patients and healthy controls [65]. However, one publication explored these clinical features in only 10 patients and was thus probably not powerful enough to show any difference. A trend for more important lipid peroxidation was found in patients, though. Of note, disorders with psychotic symptoms (e.g., schizophrenia) had reduced TAC and higher lipid peroxidation compared to healthy individuals [23,65].

#### 4.2.4. Stages of MDD

In patients with a first MDE, no strong modification of antioxidant enzymes was observed in our review (see Table 3). Decreased SOD activity was described in two articles, one in serum [28] and one in erythrocytes [66]. Another publication showed an increase in Cu/ZnSOD concentrations in the plasma of depressed patients [68]. In contrast, four publications exploring catalase, GPx, GST, and SOD, failed to show any differences between MDE patients and controls [28,38,66,67]. For oxidative damage products, one study observed an increase in plasma lipid peroxidation (through MDA levels) in MDE patients [66] while two showed no difference in plasma and serum [28,68]. In a study of serum, higher 8-OHdG levels in depressed patients were suggestive of greater DNA oxidation [59].

In recurrent MDD (see Table 4), reduced GPx activity was identified in serum and erythrocytes. Reduced SOD and Cu/ZnSOD activities and reduced MnSOD expression were equally shown [28,69,70]. Increased GR activity in erythrocytes was also observed in recurrent MDD [69]. Modifications of whole-blood GSH were also associated with recurrent MDD [69]. As expected, higher plasma concentrations of ROS (hydrogen peroxide) were also described in depressed patients [69], which agrees with the observed oxidative damage products. Indeed, higher serum and erythrocyte levels of lipid peroxidation (MDA) and DNA oxidation (8-OHdG) were found in recurrent depression [28,59,69]. Differences showed by our narrative review between first MDE and recurrent MDD suggest that patients with a chronic disorder and multiple MDEs have a more important impairment to oxidative stress and more oxidative damage.

It is notable that patients with MDD in remission have almost no modification of oxidative stress parameters compared to healthy controls (see Table 5). No differences were observed in plasma GPx and SOD activities, in plasma DNA oxidation, and in plasma TBARS and F2-isoprostanes levels [29,58]. Nonetheless, decreased serum uric acid concentrations were found in one publication [71]. These results could suggest that clinical remission in MDD could be associated with biological normalization of oxidative stress dysregulation.

#### 4.2.5. Oxidative Stress Imbalance According to Depression Severity

Patients with MDD showed differences with healthy individuals independent of depression severity (see Table 6). Indeed, patients with mild, moderate, or severe MDD had altered antioxidant enzymes (GR and SOD) and higher levels of oxidative damage products (MDA and oxLDL) compared to controls [33,72,74]. An exception with DNA oxidation was observed. Unlike patients with mild MDD, patients with severe MDD had higher serum 8-OHdG levels compared to healthy subjects [59]. Nevertheless, it is important to mention that even if modifications were observed in depressed patients, independent of MDD severity, compared to controls, significant differences between depressed patients according to the severity were also observed, notably for SOD activity, GR concentrations, and oxLDL [33,72]. Similarly, serum TAC was negatively correlated with depression severity, whereas TOS was positively correlated with severity [61]. The difference in MDA was more controversial [33].

#### 4.2.6. Child and Adolescent Depression

In child and adolescent depression, all identified studies were performed in serum (see Table 7). SOD activity was decreased among depressed youths compared to controls [75]. Similarly, oxidative damage products were increased in patients with higher MDA levels [75]. In concordance with previous results, TAC was reduced, and TOS was increased in MDD patients [75]. Whereas uric acid levels were much lower in adults suffering from MDD, however, serum uric acid concentration in young patients was unexpectedly increased compared to healthy individuals [76]. Consequently, if replicated, uric acid levels could be a novel biomarker of childhood and adolescent depression.

#### 4.2.7. Geriatric Depression

Several changes in oxidative stress biomarkers have been found in geriatric depression (see Table 7). Higher levels of free radical agents were observed, such as increased serum NO concentrations in patients with geriatric depression [78]. Antioxidant enzyme impairment, such as decreased serum SOD and plasma GPx activity, was found in older depressed patients [77,78]. Plasma GR and GST did not seem to be associated with geriatric depression [77]. Concerning non-enzymatic antioxidants, serum GSH levels were lower in depressed patients [78]. For oxidative damage products, elderly patients had more lipid peroxidation and protein oxidation, as revealed by higher levels of plasma F2-isoprotanes [77,79] and serum PCC [78]. Of note, no difference was observed in plasma TBARS and plasma PCC between patients and healthy subjects [77]. Finally, oxidative stress parameters did not appear to have different profiles between geriatric depression and unspecified MDD as compared to controls.

### 4.3. Oxidative Stress, a Target to Improve Healthcare

Given the redox imbalance identified in MDD, targeting oxidative stress for its treatment may be useful. Thus, the influence of current antidepressant drugs on peripheral oxidative stress biomarkers will be discussed in this section. As well, the use of antioxidant agents for the treatment and prevention of MDD will also be presented.

#### 4.3.1. The Antioxidant Effects of Current Treatments of MDD

Concerning antioxidant enzymes, GPx activity was assessed in seven articles after antidepressant treatment [25,26,27,28,30,31,63]. Among them, decreased activity was evidenced in two articles [28,63] and increased in one article [27]. In a meta-analysis of 3 studies, no change was highlighted in depressed patients after treatment compared to before [124]. For SOD, one publication found an increase in serum [37], four a decrease in serum and plasma [28,31,34,63], and five no difference in serum, plasma, and erythrocytes [25,26,27,33,67]. Accordingly, no significant change was evidenced in a meta-analysis of SOD after treatment in MDD patients [124]. Equally, no altered activities of catalase or GST were observed after antidepressant treatment in MDD patients [25,26,27,63,67]. For GR, a reduction was evidenced in plasma after antidepressant drugs [63], whereas no difference was found in erythrocytes [63]. Antioxidant enzymes were also assessed as pharmacogenetic biomarkers. Indeed, in a cohort of depressed patients, the SOD2 *Val16Ala* polymorphism was evaluated and was not associated with the clinical outcomes after antidepressant treatment in a cohort of depressed patients [87].

Regarding non-enzymatic antioxidants, antidepressant treatment induced increased levels of plasma ascorbic acid in MDD patients in one study [34], but three other studies did not identify differences [30,31,33]. No significant change was evidenced for vitamin E, GSH and GS-SG in plasma after treatment [30,31,33]. In one publication, serum uric acid levels increased during the follow-up of treated depressed patients [41] although this result was not consensual [31,33]. In a meta-analysis, uric acid appears to be increased in depressed patients following antidepressant treatment, suggesting that peripheral uric acid concentrations could be a useful biomarker for the management of MDD, from diagnosis to treatment efficiency [124].

For oxidative damage products, clinical studies suggest lipid peroxidation normalization after treatment. Indeed, reduced serum MDA levels were observed after antidepressant treatment in patients with MDD [34]. These results were replicated for plasma and erythrocyte MDA levels [26,63]. In agreement with these findings, a meta-analysis showed that peripheral MDA levels were reduced in MDD patients after antidepressant treatment [124]. However, no significant change was evidenced for other peripheral biomarkers of lipid peroxidation (F_2_-Isoprostanes and oxLDL) after treatment in depressed patients [30,31,58]. For nucleic acids, DNA oxidation appeared to be diminished after antidepressant treatment [58,60]. A similar result was evidenced for RNA oxidative damage. Remarkably, this decrease was more important in remitter patients [115].

For other peripheral biomarkers of oxidative stress, XO activity decreased whereas NO synthase activity increased after antidepressant treatment in patients with unipolar depression [37,50]. Additionally, as expected, TAC was enhanced while TOS was reduced after antidepressant drugs [61]. Nevertheless, these changes were not consistent in the literature [26,33,52,62].

Thus, current data suggest that antidepressant drugs are already effective antioxidant agents in the treatment of MDE in the context of MDD. Of note, physical activity and cognitive psychotherapy, well-described interventions for the treatment of MDD [14,125], are also associated with reduced oxidative stress in depressed patients [39,78].

#### 4.3.2. The Potential Effects of Antioxidant Agents in Unipolar Depression

While antidepressant drugs remain ineffective in treating resistant MDD, the identification of drugs with new mechanisms of action is necessary to improve its treatment and its prophylaxis. Several antioxidant agents have been assessed in MDD, among them Vitamin E and Zinc. In several cohorts, dietary Vitamin E intake appeared to prevent depressive symptoms and MDD in the general population [126]. However, these results were not consensual since they have not been necessarily replicated and since dietary Vitamin E intake was not correlated with plasma Vitamin E concentration [127]. A double-blind, placebo-controlled study showed that zinc supplementation, known for its antioxidant activity, was associated with better outcomes in MDD [128]. This effectiveness of zinc supplementation was associated with a normalization of antioxidant enzymes (notably Cu/ZnSOD) [40]. In addition to treating MDD, zinc intake also appeared to allow its prevention in the general population [129]. Of note, zinc concentrations were reduced in depressed patients, but increased and normalized after antidepressant treatment [124]. Data also suggest that lifestyle interventions improve depressive symptoms [130]. For example, diets with high antioxidant capacity were associated with a lower prevalence of depression [131]. Additionally, some mitochondrial modulators appear to be effective in the management of depression, notably in reestablishing the redox balance [132,133].

### 4.4. A Central Role of Oxidative Stress in the Physiopathology of MDD

**Figure 3:**
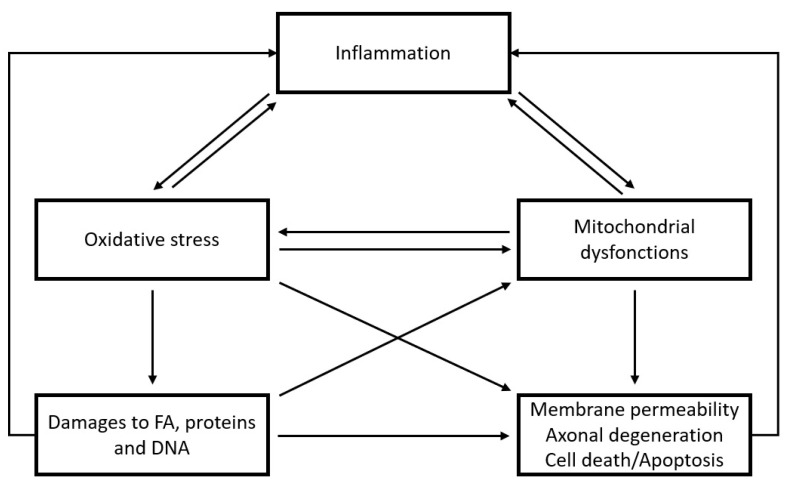
**A simplified representation of oxidative stress involvement in the physiopathology of MDD.** MDD is related to a dysregulation of oxidative stress with reduced total antioxidant capacity and increased total oxidant status. This oxidative “distress” induces a (neuro-)inflammation, mitochondrial dysfunctions and damage to fatty acids (FA), DNA and proteins. It leads to cell death, axonal degeneration, alterations of membrane permeability, and thus to neurodegenerative processes and brain modifications that are associated with unipolar depression.

Biomarkers of oxidative stress identified in this narrative review suggest a redox imbalance in MDD, with increased concentrations of ROS associated with decreased antioxidant defenses causing higher levels of oxidative damage. Oxidative stress is involved in so many signaling pathways that its impairment could lead to global and systemic disturbances [21]. Hence, oxidative stress in depression is associated with various changes, from neuroplasticity to inflammation to mitochondrial dysfunction [18,134]. Figure 3 shows a simplified representation of oxidative stress in the physiopathology of MDD.

Growing evidence suggests a link between mitochondria and MDD [134]. Mitochondria, as a direct consequence of cellular aerobic respiration, are the major endogenous source of ROS [135]. These ROS are produced particularly by the electron transport chain during oxidative phosphorylation [21]. Interestingly, the brain, due to its high energy requirement, is particularly sensitive to this redox imbalance. Consequently, among mitochondrial dysregulations described in MDD, several are induced by oxidative stress biomarkers [136]. Mitochondrial ROS cause peroxidation of mitochondrial membrane lipids and oxidation of mitochondrial DNA (mtDNA) and mitochondrial proteins [137]. Indeed, in MDD patients, a higher level of mtDNA oxidative damage was observed in leukocytes [138]. Other mtDNA impairments (e.g., mtDNA deletions and mtDNA mutations) were commonly found in depression [139,140,141]. ROS could also induce lipid membrane oxidation that could alter the permeability of both mitochondrial membranes [134,136,142]. Along these lines, altered mitochondrial membrane potential was observed in the skin fibroblast of MDD patients [143]. 

Interestingly, impairments to the electron transport chain are strongly associated with depression worsening the “oxidative distress” [134,144].

Robust evidence shows the influence of inflammation in the pathophysiology of MDD, with elevated levels of both peripheral and central proinflammatory cytokines in depressed patients [17,18]. Similarly, microglia, the resident immune cells of the brain, demonstrate, among other abnormalities, altered activation and morphological changes in depression [145,146,147]. Oxidative damage induces increased inflammation and ROS stimulates the expression of proinflammatory genes [19,21]. However, the overproduction of inflammatory cytokines is linked to reduced antioxidant activity, increasing oxidative stress. Remarkably, oxidative stress and inflammation are interrelated and interdependent mechanisms that support each other. Moreover, inflammation promotes damage to the electron transport chain in mitochondria [148].

Together, oxidative stress, inflammation, and mitochondrial dysfunction are responsible for cerebral impairments. Oxidative stress is known to cause neuronal damage, axonal degeneration, and cell death [21]. Mitochondrial alterations observed in MDD, notably concerning membrane permeability, also lead to neuronal death [149]. These neuronal degradations appear to be one of the mechanisms contributing to brain structural deficits in MDD [150]. Lower antioxidant levels were associated with reduced hippocampal volumes in MDD patients compared to healthy controls, a well-known MDD marker [151]. The effects of oxidative stress on the physiopathology of depression are principally documented through its effects on inflammation, mitochondria, and cerebral cells [18,19,21,134]. Nevertheless, a growing body of evidence shows its association and its interdependence with numerous biological pathways participating in MDD, including tryptophan-serotonin-kynurenine pathways, the regulation of glutamate and GABA, the synthesis of neurotransmitters, the hypothalamic-pituitary-adrenal axis, and cellular aging (such as telomere shortening) [18,19,144,148,150,152]. Nonetheless, to the best of our knowledge, there are no clinical trials that have explored the specific effects on cortisol levels, inflammatory processes or neurotrophic factors of antioxidant agents in patients with MDD. Ultimately, these data suggest that oxidative stress is a central piece of the depression puzzle, but further investigations should explore the causality between redox imbalance and other pathways involved in the physiopathology of MDD, including clinical trials with antioxidant agents that explore these pathways in patients with unipolar depression.

## 5. Conclusions

This narrative review focuses on oxidative stress biomarkers in MDD. It suggests a high redox imbalance in MDD as well as the potential utility of oxidative stress parameters as biomarkers for MDD management, notably in patient stratification, as specific metabolomic profiles of oxidative stress according to clinical features and disease stages are suggested. However, high heterogeneity and variability of these biomarkers was also observed, which is common in metabolomic and genetic studies [153,154]. Moreover, some of the biomarkers had a sparsity of data. Hence, in this context, replications should be performed for several parameters. Finally, this review also indicates that oxidative stress pathways could be a target of interest for the treatment of MDD.
